# Supplementary material for: XA21-mediated resistance to Xanthomonas oryzae pv. oryzae is dose dependent
Source: PeerJ. 2024 May 6;12:e17323. doi: 10.7717/peerj.17323 (PMC11080989; doi:10.7717/peerj.17323)
Supplement: Supplemental Information 1 — (A) PCR was performed with a pair of primers (LP and RP as in Table S2) that recognize genomic DNA flanking the T-DNA insertion sites on the T1 progeny of T0 events 15A, 19A, 25A, 33A, 39A, and 47A. These primers do not yield amplification product if the T-DNA insertion is homozygous as illustrated in the diagram on the top. (B) For each of the six indicated plants, two sets of primers are used to validate the genotyping results. Primer Set 1 recognizes the XA21 genomic sequence within the T-DNA and a primer that recognizes the genomic DNA near the inserted T-DNA right border (XA21Seq14F as in Table S2). Primer Set 1 was used to test the presence of the T-DNA at the expected insertion site in that line. Primer Set 2 is the same as the primers used in (A), which will not yield any amplification product only if the T-DNA insert is homozygous. T, transgenic sample; K, Kitaake control sample. [file peerj-12-17323-s001.pdf]

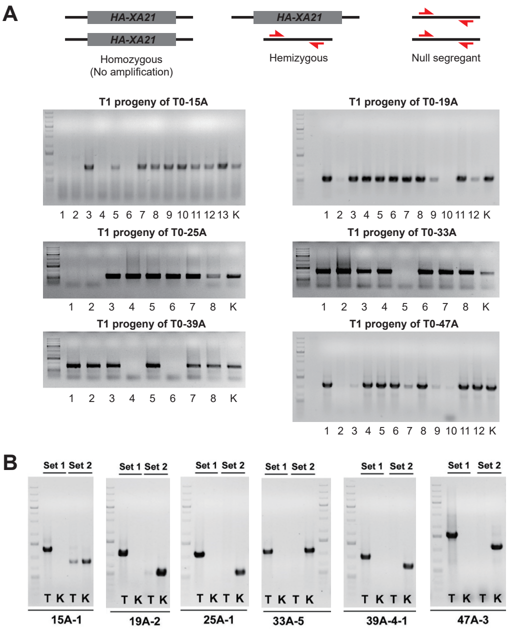

A
HA-XA21
HA-XA21
Homozygous
(No amplification)
HA-XA21
Hemizygous
Null segregant
T1 progeny of T0-15A
1 2 3 4 5 6 7 8 9 10 11 12 13 K
T1 progeny of T0-19A
1 2 3 4 5 6 7 8 9 10 11 12 K
T1 progeny of T0-25A
1 2 3 4 5 6 7 8 K
T1 progeny of T0-33A
1 2 3 4 5 6 7 8 K
T1 progeny of T0-39A
1 2 3 4 5 6 7 8 K
T1 progeny of T0-47A
1 2 3 4 5 6 7 8 9 10 11 12 K
B
Set 1 Set 2
T K T K
15A-1
19A-2
25A-1
33A-5
39A-4-1
47A-3
