# Supplementary figures and images for: XA21-mediated resistance to Xanthomonas oryzae pv. oryzae is dose dependent
Source: PeerJ. 2024 May 6;12:e17323. doi: 10.7717/peerj.17323 (PMC11080989; doi:10.7717/peerj.17323)

## Xoo inoculation

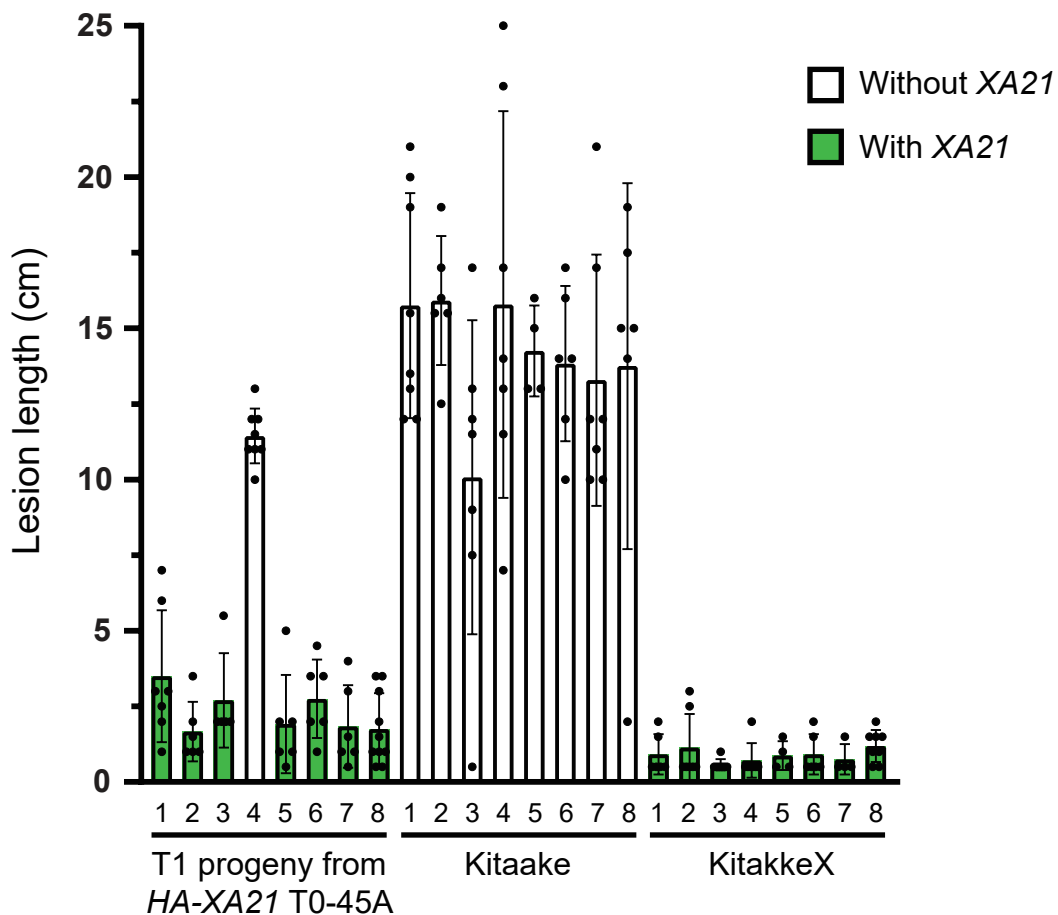

Supplement: Supplemental Information 2 — Inoculation assay of eight T1 progeny of the T0 event 45A using Xoo strain PXO99. Bars represent lesion length 14 days after clipping inoculation. White bars represent non-transgenic individuals. Filled green bars represent transgenic individuals. Kitaake was included as the susceptible control. A transgenic Kitaake line expressing XA21 under the maize Ubi-1 promoter (KitaakeX) was included as the resistant control. [file peerj-12-17323-s002.pdf]

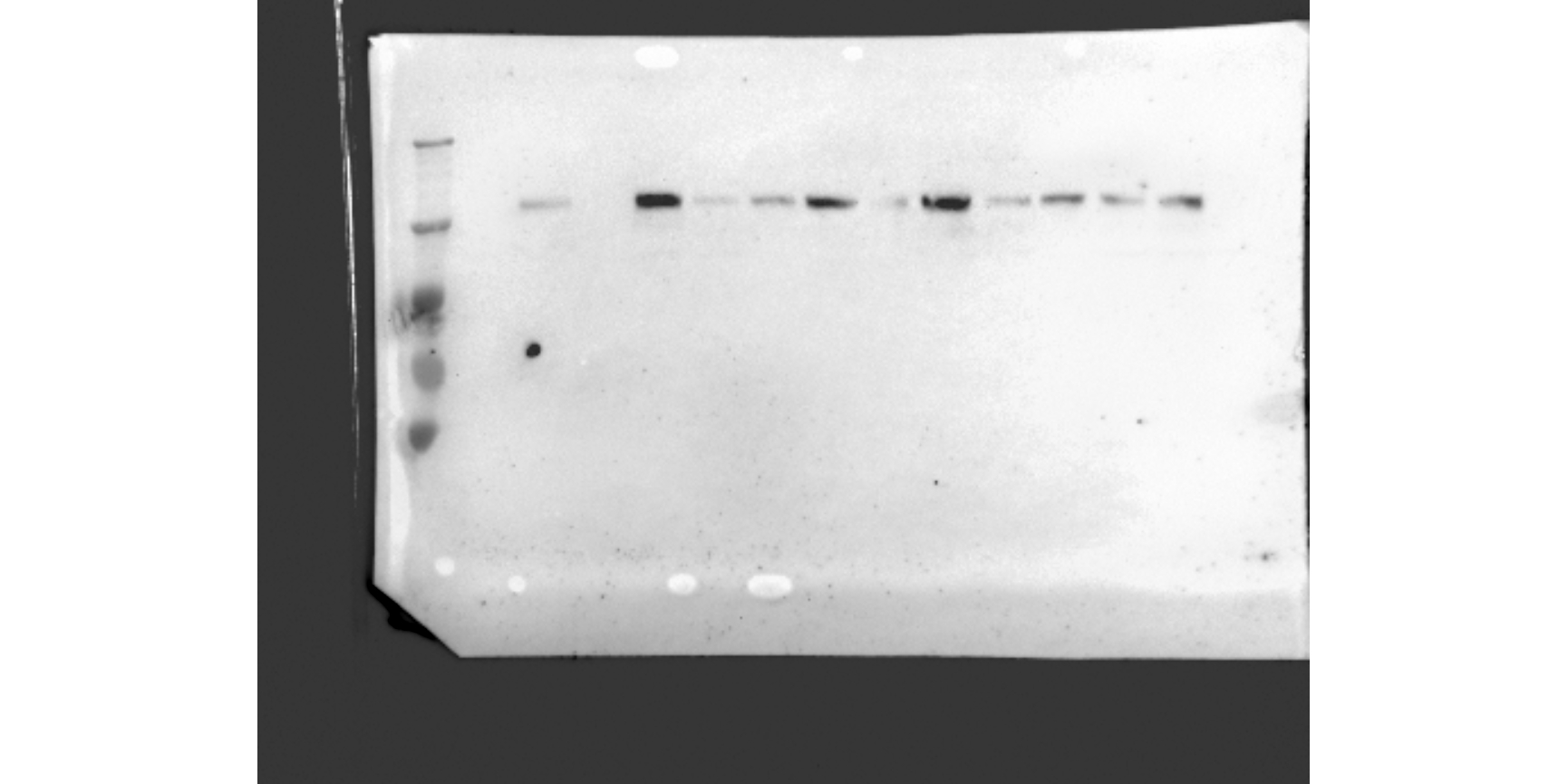

Supplement: Supplemental Information 8 [file peerj-12-17323-s008.zip › Figure 1B-1 uncropped.jpg]

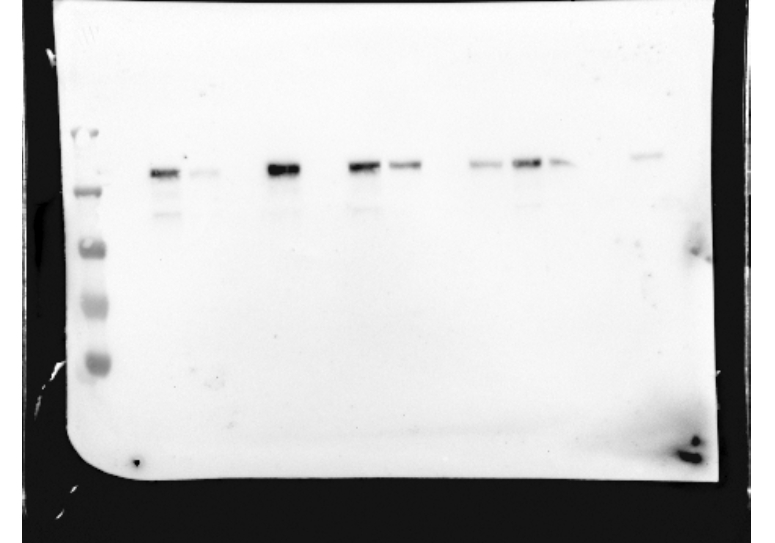

Supplement: Supplemental Information 8 [file peerj-12-17323-s008.zip › Figure 1B-2 uncropped.jpg]

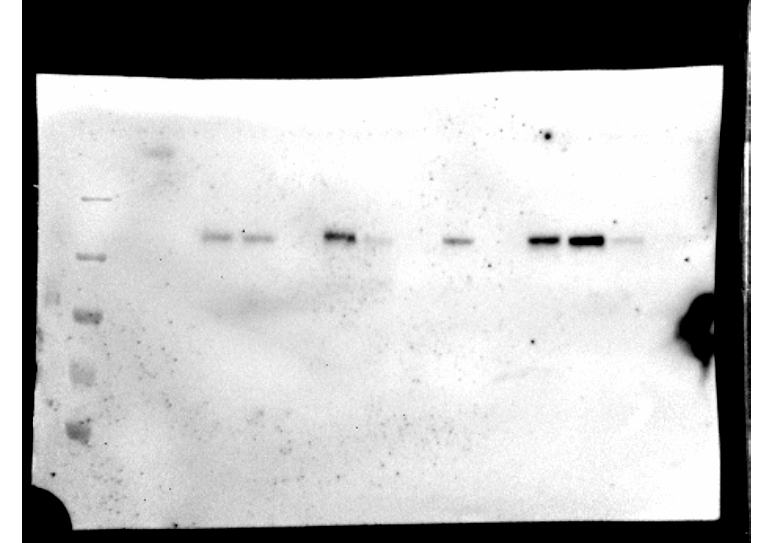

Supplement: Supplemental Information 8 [file peerj-12-17323-s008.zip › Figure 1B-3 uncropped.jpg]

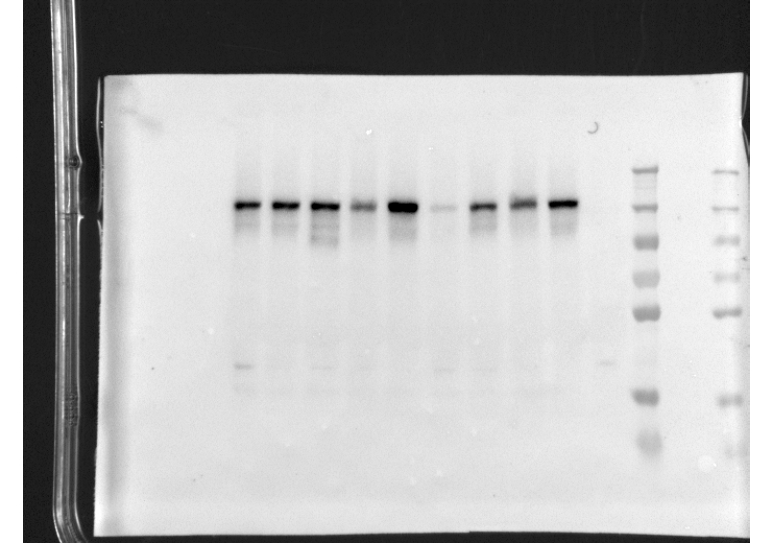

Supplement: Supplemental Information 8 [file peerj-12-17323-s008.zip › Figure 2B-1 uncropped.jpg]

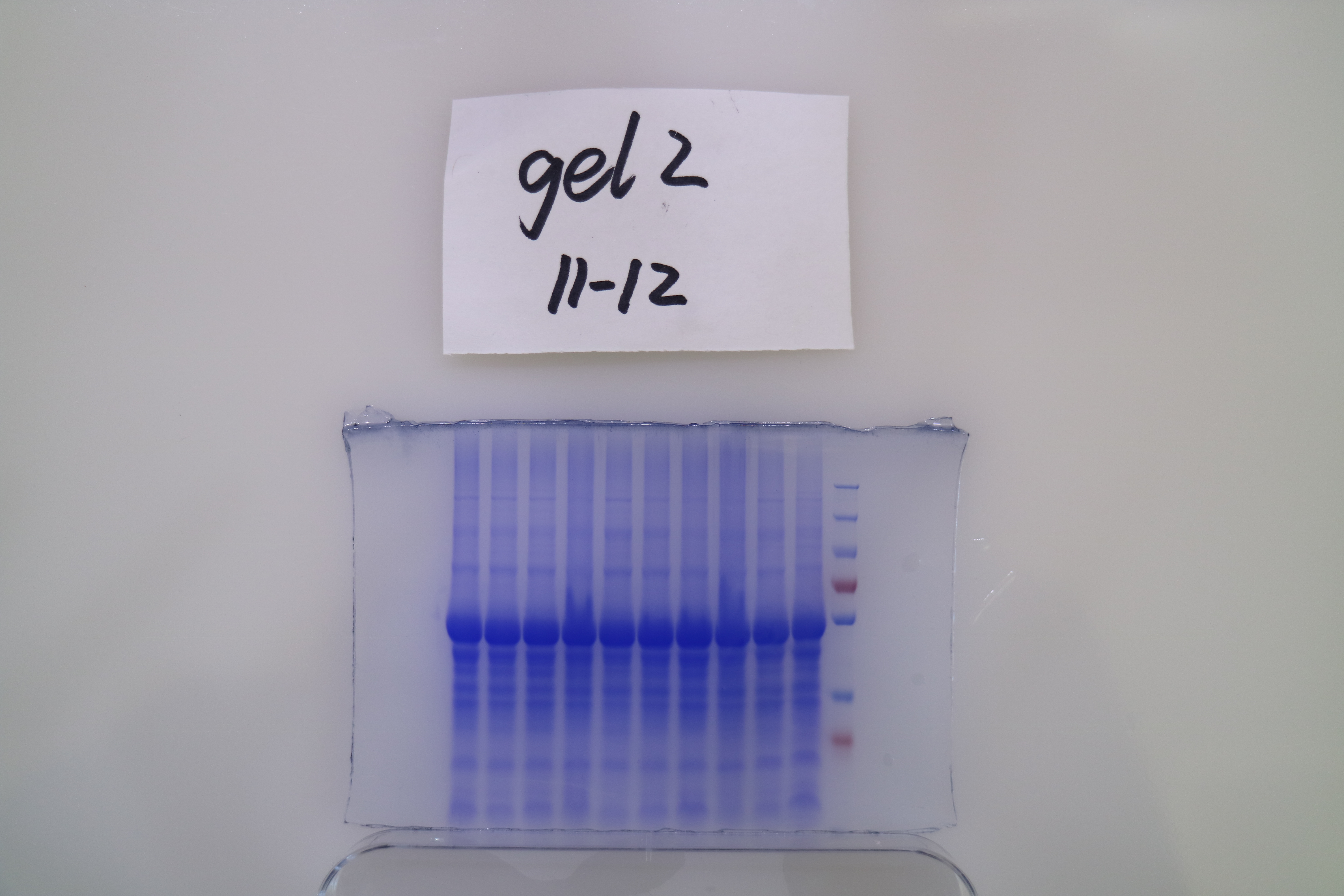

Supplement: Supplemental Information 8 [file peerj-12-17323-s008.zip › Figure 2B-2 uncropped.jpg]
