# Supplementary material for: XA21-mediated resistance to Xanthomonas oryzae pv. oryzae is dose dependent
Source: PeerJ. 2024 May 6;12:e17323. doi: 10.7717/peerj.17323 (PMC11080989; doi:10.7717/peerj.17323)
Supplement: Supplemental Information 3 [file peerj-12-17323-s003.docx]

**Table S1. Characteristics of the T-DNA insertions in 28 T0 plants expressing *HA-XA21***

| T0 plant | Mean sequencing depth | No. of T-DNA insertion sites* | Insertion site coordinate** | | No. of supporting reads | |
| --- | --- | --- | --- | --- | --- | --- |
| 1A | 64.72 | - | - | | | - |
| 4A | 28.58 | 2 | Chr.6 | 4591679 | | 16 |
|  |  |  | Chr.8 | 27063111 | | 15 |
| 5A | 5.84 | - | - | | | - |
| 7A | 29.83 | 1 | Chr.1 | 38702701 | | 18 |
| 8A | 37.12 | 2 | Chr.2 | 26163972 | | 15 |
|  |  |  | Chr.6 | 5846237 | | 15 |
| 10A | 31.41 | 1 | Chr.1 | ^‡^41450187 | | 23 |
| 11A | 31.34 | 5 | Chr.1 | 2845807 | | 18 |
|  |  |  | Chr.1 | 2757048 | | 10 |
|  |  |  | Chr.3 | 30394402 | | 14 |
|  |  |  | Chr.4 | 24019141 | | 18 |
|  |  |  | Chr.10 | 7499289 | | 11 |
| 12A | 35.97 | - | - | | | - |
| 14A | 29.13 | - | - | | | - |
| 15A | 37.99 | 1 | Chr.11 | 2352091 | | 24 |
| 16A | 63.93 | 2 | Chr.2 | 9550210 | | 27 |
|  |  |  | Chr.5 | 21901072 | | 38 |
| 19A | 61.96 | 1 | Chr.12 | 1598653 | | 49 |
| 20A | 33.7 | - | - | | | - |
| 22B | 25.91 | 1 | Chr.1 | 24122281 | | 17 |
| 24A | 31.9 | - | - | | | - |
| 25A | 34.26 | 1 | Chr.6 | 31145023 | | 28 |
| 28A | 37.9 | - | - | | | - |
| 30A | 28.88 | 2 | Chr.1 | 7333718 | | 9 |
|  |  |  | Chr.10 | 10988081 | | 25 |
| 31A | 27.07 | 1 | Chr.3 | ^§^5457747 | | 34 |
| 33A | 31.44 | 1 | Chr.1 | 42055473 | | 25 |
| 36A | 20.72 | - | - | | | - |
| 37A | 34.36 | 2 | Chr.3 | ^†^4319878 | | 4 |
|  |  |  | Chr.9 | 9886117 | | 34 |
| 39A | 53.74 | 1 | Chr.4 | 27120544 | | 17 |
| 40A | 56.05 | - | - | | | - |
| 42A | 26.63 | - | - | | | - |
| 45A | 26.02 | - | - | | | - |
| 46A | 27.36 | - | - | | | - |
| 47A | 15.87 | 1 | Chr.4 | 1983388 | | 14 |

* Only insertion sites detected in the whole-genome sequence analysis are included.

**Nipponbare reference genome.

^‡^Located within a tandem repeat region

^§^Two adjacent boundaries were detected, suggesting possible head-to-head arrangement of two T-DNA inserts.

^†^Few supporting reads.
