# Supplementary material for: XA21-mediated resistance to Xanthomonas oryzae pv. oryzae is dose dependent
Source: PeerJ. 2024 May 6;12:e17323. doi: 10.7717/peerj.17323 (PMC11080989; doi:10.7717/peerj.17323)
Supplement: Supplemental Information 4 [file peerj-12-17323-s004.docx]

| **Primer Name** | **Sequence** | **Usage in the study** |
| --- | --- | --- |
| HAXA21-PCR-I-F | cggggtacccaactttttgtgctcctattc | Fig. 1A |
| HAXA21-PCR-I-R | cgcggatccggcatagtccgggacgtcatagggatagcccgcatagtcaggaacatcgtaaggatacctgtgtgggtgccggc | Fig. 1A |
| HAXA21-PCR-II-F | cgcggatcctacccttacgacgttccagattacgctgtggtgaagctgctgctgcg | Fig. 1A |
| HAXA21-PCR-II-R | ctagctagcggtaccacactggaattctgacaatg | Fig. 1A |
| XA21Seq14F | tgcatcctttctgaagtcttga | Fig. S1A |
| 15AA-LP | gcagcgtgtcgaggtggat | Fig. S1B |
| 15AA-RP | tctctctctcactcttctcctgtc | Fig. S1B |
| 19AA-LP | atgagatattccaactgtagccttc | Fig. S1B |
| 19AA-RP | aaatatcgcggcaaaggatctacca | Fig. S1B |
| 25AA-LP | cgctgttgctttcactcccaa | Fig. S1B |
| 25AA-RP | aagatggatgatgcgtccactta | Fig. S1B |
| 33AA-LP | cgggcacggggaaagaaactt | Fig. S1B |
| 33AA-RP | gcgcacccatgatactcctact | Fig. S1B |
| 39AA-LP | ccacggacagctactgtgacgt | Fig. S1B |
| 39AA-RP | gcgggcatactagacggtatgt | Fig. S1B |
| 47AA-LP | tgccgatctgcaccgtatgc | Fig. S1B |
| 47AA-RP | agcagctcatctatcatgttctcc | Fig. S1B |

**Table S2. Primers used in this study**
