# Supplementary material for: XA21-mediated resistance to Xanthomonas oryzae pv. oryzae is dose dependent
Source: PeerJ. 2024 May 6;12:e17323. doi: 10.7717/peerj.17323 (PMC11080989; doi:10.7717/peerj.17323)
Supplement: Supplemental Information 5 [file peerj-12-17323-s005.docx]

**Table S3. Summary of the resequencing data for 28 individual *HA-XA21* T0 transgenic plants.** Sample names, accession numbers, and the mean sequencing depth are provided.

| **Sample** | **Accession number** | **Mean sequencing depth** |
| --- | --- | --- |
| 3xHA-XA21_47A | SRP296494 | 15.87 |
| 3xHA-XA21_45A | SRP296493 | 26.02 |
| 3xHA-XA21_46A | SRP296492 | 27.36 |
| 3xHA-XA21_40A | SRP296490 | 56.05 |
| 3xHA-XA21_42A | SRP296489 | 26.63 |
| 3xHA-XA21_39A | SRP296488 | 53.74 |
| 3xHA-XA21_37A | SRP296487 | 34.36 |
| 3xHA-XA21_33A | SRP296485 | 31.44 |
| 3xHA-XA21_36A | SRP296484 | 20.72 |
| 3xHA-XA21_30A | SRP296483 | 28.88 |
| 3xHA-XA21_31A | SRP296482 | 27.07 |
| 3xHA-XA21_28A | SRP296481 | 37.9 |
| 3xHA-XA21_25A | SRP296480 | 34.26 |
| 3xHA-XA21_24A | SRP296479 | 31.9 |
| 3xHA-XA21_20A | SRP296478 | 33.7 |
| 3xHA-XA21_22B | SRP296477 | 25.91 |
| 3xHA-XA21_16A | SRP296476 | 63.93 |
| 3xHA-XA21_19A | SRP296475 | 61.96 |
| 3xHA-XA21_15A | SRP296474 | 37.99 |
| 3xHA-XA21_14A | SRP296473 | 29.13 |
| 3xHA-XA21_12A | SRP296471 | 35.97 |
| 3xHA-XA21_8A | SRP296470 | 37.12 |
| 3xHA-XA21_11A | SRP296469 | 31.34 |
| 3xHA-XA21_7A | SRP296468 | 29.83 |
| 3xHA-XA21_10A | SRP296467 | 31.41 |
| 3xHA-XA21_4A | SRP296466 | 28.58 |
| 3xHA-XA21_5A | SRP296465 | 5.84 |
| 3xHA-XA21_1A | SRP296463 | 64.72 |
