# Supplementary material for: XA21-mediated resistance to Xanthomonas oryzae pv. oryzae is dose dependent
Source: PeerJ. 2024 May 6;12:e17323. doi: 10.7717/peerj.17323 (PMC11080989; doi:10.7717/peerj.17323)
Supplement: Supplemental Information 6 [file peerj-12-17323-s006.docx]

**Supplemental Data 1. Full sequence of the pCambia2300-HA-XA21 construct.**

catgccaaccacagggttcccctcgggatcaaagtactttgatccaacccctccgctgctatagtgcagtcggcttctgacgttcagtgcagccgtcttctgaaaacgacatgtcgcacaagtcctaagttacgcgacaggctgccgccctgcccttttcctggcgttttcttgtcgcgtgttttagtcgcataaagtagaatacttgcgactagaaccggagacattacgccatgaacaagagcgccgccgctggcctgctgggctatgcccgcgtcagcaccgacgaccaggacttgaccaaccaacgggccgaactgcacgcggccggctgcaccaagctgttttccgagaagatcaccggcaccaggcgcgaccgcccggagctggccaggatgcttgaccacctacgccctggcgacgttgtgacagtgaccaggctagaccgcctggcccgcagcacccgcgacctactggacattgccgagcgcatccaggaggccggcgcgggcctgcgtagcctggcagagccgtgggccgacaccaccacgccggccggccgcatggtgttgaccgtgttcgccggcattgccgagttcgagcgttccctaatcatcgaccgcacccggagcgggcgcgaggccgccaaggcccgaggcgtgaagtttggcccccgccctaccctcaccccggcacagatcgcgcacgcccgcgagctgatcgaccaggaaggccgcaccgtgaaagaggcggctgcactgcttggcgtgcatcgctcgaccctgtaccgcgcacttgagcgcagcgaggaagtgacgcccaccgaggccaggcggcgcggtgccttccgtgaggacgcattgaccgaggccgacgccctggcggccgccgagaatgaacgccaagaggaacaagcatgaaaccgcaccaggacggccaggacgaaccgtttttcattaccgaagagatcgaggcggagatgatcgcggccgggtacgtgttcgagccgcccgcgcacgtctcaaccgtgcggctgcatgaaatcctggccggtttgtctgatgccaagctggcggcctggccggccagcttggccgctgaagaaaccgagcgccgccgtctaaaaaggtgatgtgtatttgagtaaaacagcttgcgtcatgcggtcgctgcgtatatgatgcgatgagtaaataaacaaatacgcaaggggaacgcatgaaggttatcgctgtacttaaccagaaaggcgggtcaggcaagacgaccatcgcaacccatctagcccgcgccctgcaactcgccggggccgatgttctgttagtcgattccgatccccagggcagtgcccgcgattgggcggccgtgcgggaagatcaaccgctaaccgttgtcggcatcgaccgcccgacgattgaccgcgacgtgaaggccatcggccggcgcgacttcgtagtgatcgacggagcgccccaggcggcggacttggctgtgtccgcgatcaaggcagccgacttcgtgctgattccggtgcagccaagcccttacgacatatgggccaccgccgacctggtggagctggttaagcagcgcattgaggtcacggatggaaggctacaagcggcctttgtcgtgtcgcgggcgatcaaaggcacgcgcatcggcggtgaggttgccgaggcgctggccgggtacgagctgcccattcttgagtcccgtatcacgcagcgcgtgagctacccaggcactgccgccgccggcacaaccgttcttgaatcagaacccgagggcgacgctgcccgcgaggtccaggcgctggccgctgaaattaaatcaaaactcatttgagttaatgaggtaaagagaaaatgagcaaaagcacaaacacgctaagtgccggccgtccgagcgcacgcagcagcaaggctgcaacgttggccagcctggcagacacgccagccatgaagcgggtcaactttcagttgccggcggaggatcacaccaagctgaagatgtacgcggtacgccaaggcaagaccattaccgagctgctatctgaatacatcgcgcagctaccagagtaaatgagcaaatgaataaatgagtagatgaattttagcggctaaaggaggcggcatggaaaatcaagaacaaccaggcaccgacgccgtggaatgccccatgtgtggaggaacgggcggttggccaggcgtaagcggctgggttgtctgccggccctgcaatggcactggaacccccaagcccgaggaatcggcgtgacggtcgcaaaccatccggcccggtacaaatcggcgcggcgctgggtgatgacctggtggagaagttgaaggccgcgcaggccgcccagcggcaacgcatcgaggcagaagcacgccccggtgaatcgtggcaagcggccgctgatcgaatccgcaaagaatcccggcaaccgccggcagccggtgcgccgtcgattaggaagccgcccaagggcgacgagcaaccagattttttcgttccgatgctctatgacgtgggcacccgcgatagtcgcagcatcatggacgtggccgttttccgtctgtcgaagcgtgaccgacgagctggcgaggtgatccgctacgagcttccagacgggcacgtagaggtttccgcagggccggccggcatggccagtgtgtgggattacgacctggtactgatggcggtttcccatctaaccgaatccatgaaccgataccgggaagggaagggagacaagcccggccgcgtgttccgtccacacgttgcggacgtactcaagttctgccggcgagccgatggcggaaagcagaaagacgacctggtagaaacctgcattcggttaaacaccacgcacgttgccatgcagcgtacgaagaaggccaagaacggccgcctggtgacggtatccgagggtgaagccttgattagccgctacaagatcgtaaagagcgaaaccgggcggccggagtacatcgagatcgagctagctgattggatgtaccgcgagatcacagaaggcaagaacccggacgtgctgacggttcaccccgattactttttgatcgatcccggcatcggccgttttctctaccgcctggcacgccgcgccgcaggcaaggcagaagccagatggttgttcaagacgatctacgaacgcagtggcagcgccggagagttcaagaagttctgtttcaccgtgcgcaagctgatcgggtcaaatgacctgccggagtacgatttgaaggaggaggcggggcaggctggcccgatcctagtcatgcgctaccgcaacctgatcgagggcgaagcatccgccggttcctaatgtacggagcagatgctagggcaaattgccctagcaggggaaaaaggtcgaaaaggtctctttcctgtggatagcacgtacattgggaacccaaagccgtacattgggaaccggaacccgtacattgggaacccaaagccgtacattgggaaccggtcacacatgtaagtgactgatataaaagagaaaaaaggcgatttttccgcctaaaactctttaaaacttattaaaactcttaaaacccgcctggcctgtgcataactgtctggccagcgcacagccgaagagctgcaaaaagcgcctacccttcggtcgctgcgctccctacgccccgccgcttcgcgtcggcctatcgcggccgctggccgctcaaaaatggctggcctacggccaggcaatctaccagggcgcggacaagccgcgccgtcgccactcgaccgccggcgcccacatcaaggcaccctgcctcgcgcgtttcggtgatgacggtgaaaacctctgacacatgcagctcccggagacggtcacagcttgtctgtaagcggatgccgggagcagacaagcccgtcagggcgcgtcagcgggtgttggcgggtgtcggggcgcagccatgacccagtcacgtagcgatagcggagtgtatactggcttaactatgcggcatcagagcagattgtactgagagtgcaccatatgcggtgtgaaataccgcacagatgcgtaaggagaaaataccgcatcaggcgctcttccgcttcctcgctcactgactcgctgcgctcggtcgttcggctgcggcgagcggtatcagctcactcaaaggcggtaatacggttatccacagaatcaggggataacgcaggaaagaacatgtgagcaaaaggccagcaaaaggccaggaaccgtaaaaaggccgcgttgctggcgtttttccataggctccgcccccctgacgagcatcacaaaaatcgacgctcaagtcagaggtggcgaaacccgacaggactataaagataccaggcgtttccccctggaagctccctcgtgcgctctcctgttccgaccctgccgcttaccggatacctgtccgcctttctcccttcgggaagcgtggcgctttctcatagctcacgctgtaggtatctcagttcggtgtaggtcgttcgctccaagctgggctgtgtgcacgaaccccccgttcagcccgaccgctgcgccttatccggtaactatcgtcttgagtccaacccggtaagacacgacttatcgccactggcagcagccactggtaacaggattagcagagcgaggtatgtaggcggtgctacagagttcttgaagtggtggcctaactacggctacactagaaggacagtatttggtatctgcgctctgctgaagccagttaccttcggaaaaagagttggtagctcttgatccggcaaacaaaccaccgctggtagcggtggtttttttgtttgcaagcagcagattacgcgcagaaaaaaaggatctcaagaagatcctttgatcttttctacggggtctgacgctcagtggaacgaaaactcacgttaagggattttggtcatgcattctaggtactaaaacaattcatccagtaaaatataatattttattttctcccaatcaggcttgatccccagtaagtcaaaaaatagctcgacatactgttcttccccgatatcctccctgatcgaccggacgcagaaggcaatgtcataccacttgtccgccctgccgcttctcccaagatcaataaagccacttactttgccatctttcacaaagatgttgctgtctcccaggtcgccgtgggaaaagacaagttcctcttcgggcttttccgtctttaaaaaatcatacagctcgcgcggatctttaaatggagtgtcttcttcccagttttcgcaatccacatcggccagatcgttattcagtaagtaatccaattcggctaagcggctgtctaagctattcgtatagggacaatccgatatgtcgatggagtgaaagagcctgatgcactccgcatacagctcgataatcttttcagggctttgttcatcttcatactcttccgagcaaaggacgccatcggcctcactcatgagcagattgctccagccatcatgccgttcaaagtgcaggacctttggaacaggcagctttccttccagccatagcatcatgtccttttcccgttccacatcataggtggtccctttataccggctgtccgtcatttttaaatataggttttcattttctcccaccagcttatataccttagcaggagacattccttccgtatcttttacgcagcggtatttttcgatcagttttttcaattccggtgatattctcattttagccatttattatttccttcctcttttctacagtatttaaagataccccaagaagctaattataacaagacgaactccaattcactgttccttgcattctaaaaccttaaataccagaaaacagctttttcaaagttgttttcaaagttggcgtataacatagtatcgacggagccgattttgaaaccgcggtgatcacaggcagcaacgctctgtcatcgttacaatcaacatgctaccctccgcgagatcatccgtgtttcaaacccggcagcttagttgccgttcttccgaatagcatcggtaacatgagcaaagtctgccgccttacaacggctctcccgctgacgccgtcccggactgatgggctgcctgtatcgagtggtgattttgtgccgagctgccggtcggggagctgttggctggctggtggcaggatatattgtggtgtaaacaaattgacgcttagacaacttaataacacattgcggacgtttttaatgtactgaattaacgccgaattaattcgggggatctggattttagtactggattttggttttaggaattagaaattttattgatagaagtattttacaaatacaaatacatactaagggtttcttatatgctcaacacatgagcgaaaccctataggaaccctaattcccttatctgggaactactcacacattattatggagaaactcgagcttgtcgatcgactctagctagaggatcgatccgaaccccagagtcccgctcagaagaactcgtcaagaaggcgatagaaggcgatgcgctgcgaatcgggagcggcgataccgtaaagcacgaggaagcggtcagcccattcgccgccaagctcttcagcaatatcacgggtagccaacgctatgtcctgatagcggtccgccacacccagccggccacagtcgatgaatccagaaaagcggccattttccaccatgatattcggcaagcaggcatcgccatgtgtcacgacgagatcctcgccgtcgggcatgcgcgccttgagcctggcgaacagttcggctggcgcgagcccctgatgctcttcgtccagatcatcctgatcgacaagaccggcttccatccgagtacgtgctcgctcgatgcgatgtttcgcttggtggtcgaatgggcaggtagccggatcaagcgtatgcagccgccgcattgcatcagccatgatggatactttctcggcaggagcaaggtgagatgacaggagatcctgccccggcacttcgcccaatagcagccagtcccttcccgcttcagtgacaacgtcgagcacagctgcgcaaggaacgcccgtcgtggccagccacgatagccgcgctgcctcgtcctggagttcattcagggcaccggacaggtcggtcttgacaaaaagaaccgggcgcccctgcgctgacagccggaacacggcggcatcagagcagccgattgtctgttgtgcccagtcatagccgaatagcctctccacccaagcggccggagaacctgcgtgcaatccatcttgttcaatccccatggtcgatcgacagatctgcgaaagctcgagagagatagatttgtagagagagactggtgatttcagcgtgtcctctccaaatgaaatgaacttccttatatagaggaaggtcttgcgaaggatagtgggattgtgcgtcatcccttacgtcagtggagatatcacatcaatccacttgctttgaagacgtggttggaacgtcttctttttccacgatgctcctcgtgggtgggggtccatctttgggaccactgtcggcagaggcatcttgaacgatagcctttcctttatcgcaatgatggcatttgtaggtgccaccttccttttctactgtccttttgatgaagtgacagatagctgggcaatggaatccgaggaggtttcccgatattaccctttgttgaaaagtctcaatagccctttggtcttctgagactgtatctttgatattcttggagtagacgagagtgtcgtgctccaccatgttatcacatcaatccacttgctttgaagacgtggttggaacgtcttctttttccacgatgctcctcgtgggtgggggtccatctttgggaccactgtcggcagaggcatcttgaacgatagcctttcctttatcgcaatgatggcatttgtaggtgccaccttccttttctactgtccttttgatgaagtgacagatagctgggcaatggaatccgaggaggtttcccgatattaccctttgttgaaaagtctcaatagccctttggtcttctgagactgtatctttgatattcttggagtagacgagagtgtcgtgctccaccatgttggcaagctgctctagccaatacgcaaaccgcctctccccgcgcgttggccgattcattaatgcagctggcacgacaggtttcccgactggaaagcgggcagtgagcgcaacgcaattaatgtgagttagctcactcattaggcaccccaggctttacactttatgcttccggctcgtatgttgtgtggaattgtgagcggataacaatttcacacaggaaacagctatgacatgattacgaattcgagctcggtacccaactttttgtgctcctattcgcaacctgggtacaaggacgttttgtggtatgctaacatccggtccaatttctagaccaactttttcacttttgcagtcattttttacatcgcacaacatttgaccaagattatccgtaccgccgttttcttcaacagctctgtccgcctcgcctattgtattttctgcaaatccatcatattgagcccagtctgaaatgttgtcgtcttcttattaattttcttctattgcaacccctaactctccgtgcaaggtctaacaattatagccaggtatgaatcccgattccaacaagtggatatgaagacttcttagatgtagaatactccttctggtttttgcacttttgcatggacaacatataaaacccttaggcttgtgggcattggccacactcaaaaaataatgcacgtcatcaataaactccttcgaccgtcgttctgcagtgtacatccattaccgattcatctacattaggagataataattgtaaaggaagtccacaaaagtgaaactacttaaataatcatataataatttaaaatatcataattaaattgaaaactgacggttttaatgtattcttcttatttctaacgattttaacgagttttaaatggacttaatcggagtcatgattaactatttataaattttatctgtctcaataaattgtaaatatatttttccatgtattatccttgtttaattatttttaaaagttctaaacatatttttaatgcattctacttatttctaactattttaaagattttcaaatggacttagttttctatttttattcttcattttctatatttgccctttgttgtctctttttaacaattttatacaaatatttataattttattaagtaccctaattttccctaaacaatttttctctctcatcgtatttccatatatctttttgagataataatggatataaacatagctagaaatgtaaatgttcaccttgcatcaataggggatgaagttgctaaccttttagatctcctcgatttgtataatataaccaaaatattttcaccaaaaatttcgttaaacatccgagatatttgttgtttttgccgatcgagcaaagattagtagtccagcagtgtctgcaccaccaccatcgtgataatgcatcttgtgtgttattcttgatgagaaaatacgtagtgaaaaccacatatgtggtggaaacttagaaactaccgttagatcgagaaatggatgtccaagattcgtccacgtcaccaagagataaaatttagctcgcagattcacttatgagttaaaattttaatgagagttaaattttaactcatgttgatgtggacgaatatcggacatccatttctcgatccaacgatagcttccaagtttccactacatatgtggtttgcactatatattttcccattcttgattatgtgtttgagagcagctagcacaaagagaaaaaaaagcatcgtttttcacgcgtatgttttcagaactgttaaatggtgtgttttttgaaaaaactttctatagaaaagtttctttaaaaaatatattaatctattttttaagtttaaaataattactacttaattaattatacactaacagcttatttcgttctacgtatcttgtcaattttcgctattcctttcttctcaaacacggcattggatgctctcatagcacttgctcgttcggatagaagacttgacgaagacgaccgctacaacttggtgtgttatatcgtgctttgtttagcataatcattacatatattccatgccgaagtgccgacgatgagaccgtgttcgatgcatctttgtatggcatctagggacaaagagcatagagtccctaccatagtaccagctcgcgcagaagacttgacgagaagaccgactgctacaccttggtgtgtaataatatcgtgttgtgtgtaccatgcatactcctttaaaacaaataatggtggtaacagtaaatctgtcatcccacccactctcattgtaaattttgcaagttctcacttgaacttcttaatactccatccgtttgcgtgtgttctttcagaatttgcgtgagcactttttcttctatataatctgtctagtccatgagctaaaccaacatctctcgctgtcttgccttgcacttctgcacgatgatatcactcccattattgctcttcgtcctgttgttctctgcgctgctgctctgcccttcaagcagtgacgacgatggtgatgctgccggcgacgaactcgcgctgctctctttcaagtcatccctgctataccaggggggccagtcgctggcatcttggaacacgtccggccacggccagcactgcacatgggtgggtgttgtgtgcggccgccgccgccgccggcacccacacaggtatccttacgatgttcctgactatgcgggctatccctatgacgtcccggactatgccggatcctacccttacgacgttccagattacgctgtggtgaagctgctgctgcgctcctccaacctgtccgggatcatctcgccgtcgctcggcaacctgtccttcctcagggagctggacctcggcgacaactacctctccggcgagataccaccggagctcagccgtctcagcaggcttcagctgctggagctgagcgataactccatccaagggagcatccccgcggccattggagcatgcaccaagttgacatcgctagacctcagccacaaccaactgcgaggtatgatcccacgtgagattggtgccagcttgaaacatctctcgaatttgtacctttacaaaaatggtttgtcaggagagattccatccgctttgggcaatctcactagcctccaggagtttgatttgagcttcaacagattatcaggagctataccttcatcactggggcagctcagcagtctattgactatgaatttgggacagaacaatctaagtgggatgatccccaattctatctggaacctttcgtctctaagagcgtttagtgtcagagaaaacaagctaggtggtatgatccctacaaatgcattcaaaacccttcacctcctcgaggtgatagatatgggcactaaccgtttccatggcaaaatccctgcctcagttgctaatgcttctcatttgacagtgattcagatttatggcaacttgttcagtggaattatcacctcggggtttggaaggttaagaaatctcacagaactgtatctctggagaaatttgtttcaaactagagaacaagatgattgggggttcatttctgacctaacaaattgctccaaattacaaacattgaacttgggagaaaataacctggggggagttcttcctaattcgttttccaatctttccacttcgcttagttttcttgcacttgaattgaataagatcacaggaagcattccgaaggatattggcaatcttattggcttacaacatctctatctctgcaacaacaatttcagagggtctcttccatcatcgttgggcaggcttaaaaacttaggcattctactcgcctacgaaaacaacttgagcggttcgatcccgttggccataggaaatcttactgaacttaatatcttactgctcggcaccaacaaattcagtggttggataccatacacactctcaaacctcacaaacttgttgtcattaggcctttcaactaataaccttagtggtccaatacccagtgaattattcaatattcaaacactatcaataatgatcaatgtatcaaaaaataacttggagggatcaataccacaagaaatagggcatctcaaaaatctagtagaatttcatgcagaatcgaatagattatcaggtaaaatccctaacacgcttggtgattgccagctcttacggtatctttatctgcaaaataatttgttatctggtagcatcccatcagccttgggtcagctgaaaggtctcgaaactcttgatctctcaagcaacaatttgtcaggccagatacccacatccttagcagatattactatgcttcattccttgaacctttctttcaacagctttgtgggggaagtgccaaccattggtgctttcgcagctgcatccgggatctcaatccaaggcaatgccaaactctgtggtggaatacctgatctacatctgcctcgatgttgtccattactagagaacagaaaacatttcccagttctacctatttctgtttctctggccgcagcactggccatcctctcatcactctacttgcttataacctggcacaagagaactaaaaagggagccccttcaagaacttccatgaaaggccacccattggtctcttattcgcagttggtaaaagcaacagatggtttcgcgccgaccaatttgttgggttctggatcatttggctcagtatacaaaggaaagcttaatatccaagatcatgttgcagtgaaggtactaaagcttgaaaatcctaaggcgctcaagagtttcactgccgaatgtgaagcactacgaaatatgcgacatcgaaatcttgtcaagatagttacaatttgctcgagcattgataacagagggaacgatttcaaagcaattgtgtatgacttcatgcccaacggcagtctggaagattggatacaccctgaaacaaatgatcaagcagaccagaggcacttgaatctgcatcgaagagtgaccatactacttgatgttgcctgcgcactggactatcttcaccgccatggccctgaacctgttgtacactgtgatattaaatcaagcaatgtgctgttagattctgatatggtagcccatgttggagattttgggcttgcaagaatacttgttgatgggacctcattgatacaacagtcaacaagctcgatgggatttatagggacaattggctatgcagcaccaggtcagcaagtccttccagtattttgcattttctgatctctagtgctatatgaaatagtttttacctctagtgaaactgatggagaatataagtaattaattgaactaattaaattgcacaaaaataagattatttgccatatctattcagatgctaaatatagctagttcatagaggtacagatttttttatataggactctagagctaccacacactcaaatcaaattatgggtgttttctgctctacactgcaatatgaaatgattattacttctacatgaactgatggaggagtttcagaaggatcaaatttgagtaaatttttcaattctacatttaagaaacacttttttttcatatgctagttacatttttttatttcacgagcttacattgaccatgaaaaatacttggcactacttactaattcccacatggaggtagtgaaaataatatagatacaaaaacgaaatatcctatgttgtgtgatatactataatcacaatgaacacaaacaggattcgtacaaaagtaattagccatcatagcaactgattgcttggggtaactgtatagcacaatcataccaaatttctttagatatgtatctgtaaattagattcttaaagttaaatatgaaatttcattggtatttatgtttctttatataataaaaattaatccagcctttgcatctatcatttgtccagacatccttgttatttgtgatatttaacacgtaaatttacataattatacatccaagttctttttatttaacactgtaaatttcaaatcgtacatgttataaagaatgtactatatttcctgctcaaacagagtatggcgttgggctcattgcatcaacgcatggagatatttacagctatggaattctagtgctggaaatagtaaccgggaagcggccaactgacagtacattcagacccgatttgggcctccgtcagtacgttgaactgggcctacatggcagagtgacggatgttgttgacacgaagctcattttggattctgagaactggctgaacagtacaaataattctccatgtagaagaatcactgaatgcattgtttggctgcttagacttgggttgtcttgctctcaggaattgccatcgagtagaacgccaaccggagatatcatcgacgaactgaatgccatcaaacagaatctctccggattgtttccagtgtgtgaaggtgggagccttgaattctgatgttatgtctcgtaatgttttattgccacacttcagatcgacttctgcagtggtatctaccacacgatcactaaagtcaccgtggctatttcctgatccagcatatctgatcatgcatgttctgtgttgtatacctgtattttactctgaattgccacaccgcaaccctgcctctgtttgtttggtatacaaaagatagtgatgagtttattgttttaggggcttcctagttggcgcgtgtgcatgccggcatgcacgcagcccgagggtgggtttcttttttttccattgttattccgttgctttttttcaccacggtagatttttttttccggatttccattttttccgttgtttttctctatcgcttatgttggcggatttttttccgtggttttctttccgaagacgagtatatctaacgtaactaacatgttacttttagataacgatggttattaagataagatttttctctggaagatttttgtaagtaacagattgaaaacaaatctatacgtgaggtcaaattttgaaaactttcaatctagatttaaaagcttttcaactcaaaatttgaatttttgaagtgaaaatttgaatactttcaaaaattactagtaatcgacaaaaaaaatatggaaatggaaacggaaatagttttgctgttataccgatcgtttccatatttaccgtattcttatagaaattaccgtttcttataatatggtaattaccgtatttctaaatatgttgatatttatagggcatgtctctacttgactcacagtttagagattgattgactatttaatcaaatccctaacttgattgcatggctaaaatggagttgatttctaatttatatagtatagcttgaatttatttgtaaatataacatacttatgtaaagttaaatatatgttttctatagtttaatgtttctgtatttgttaccggttttcgatctgtaccgacatatttccatcagtattattccatttccggttttccgatatttccgatatcgttttcgtttccgactttaccgttttcgatttcatttccgagaaaaatatgattatggaaatggtcgaggctgttttccgatcgtttccgaccgttttcatccctacccgtagtaataatatataacattttatctctaatctttctctctctcatatcaatgaataatcgctaagagactgctattaacaaggcttatatatatatatgccgtcgatcagtcattttgaaacggcccacttcttttccatctatatgcattcatgaaatacatggtatatcccatcgatcggacatcacctgttagcgcgtacgccatcgtcgtcatcaacctagctagggcaaacgcaccttgctgagctccgatcctccgatcgccaccatcaccaatgaacaagctgctgcggcctctcggtggcctgaggttgctcaaccgagaagaacatccgttccgatgcttctcctcctccatcgatctcgtcttcccaggtcgccgccgccgccacatggcaaccaccgtgacccacccgccgccgacggaatccgctggttcgacggcggcggccgcgactgctgacccggcctcggtgatgctggaacattggggctgcctcaggggctccacgccggcgaacgtagtcgccgacgacaacaccgccgcggagtcccgcacctcccgcggccaacccctccgcgtcgccctcgcccgcgcgtcgccgccggcgatctccttcatctgcttcgatcgcggggatgatggctacgtcatcgcggctcacggcgactctgtcctcttccggatgagttggaacgactacttcgtctacatggccgccggcggccgccgtcgctgacgctgctccccgtctgcgacatccccatgaacgagcgctgctgggtcagcaaggaccgtttcaaggacagcttccacaccacgggccgggagttcgaccagcaggacaccggcatcctgcgcctccgcggcgacgacggcggcgaggaggcgccgcctctagtggcgcagctccagatcgcgcacgagccgccgttcgacacggccgagctctgcgtgctccgccccggccacggcgagtgggagctcaagatggcggtgcccatcgtccaccatgacagcttgaatcttataacaaaccttgctgaagctgacaaatcctagcccccagccatgaagttggaaaaatcaatttccgattacacaaattggttaatacgcaaccatttagtgctcttaacatgaccaggttttacatgttcgttcggctcttagaatctgacaataccttatctgctctgggcgtccccagccgaaattccattagttttctcggaggcttgtcagaacagcgtaaagggacaataggactgccttcaagatgaggcgatataagacgggatcaacagacaaatattgcacatataaaacttacagaagttgatgtagatgatgagacgaccaccacactaggcaaagaccaggtgtatagttgtactcaacaaatcgcagaggtagtgagagatcgctacgatctactggtcaaagatcaggtgtaggcgtattctcgatcacctgaagaagaatctttaggtgttgagagatcgctactatctactggtcaaaactagtaaaaaaacctcatagagatcggcactataggtgccggaacagctaaaaccggcacctataatacttttccctcctccgtggactcaaagcacgtaaaaccgacacctttaagcaactataggtgccggttctaaagaagaaccgacacctatagtataggtgctggttttttaaaaaaacctgacacctttaatataatataggtgtcggttcttctttaaaaccgacaccaataataaattatacgtgtcggttttttaataaaaccggcacctatccaaaccgatcctagctgtcgagtcgagccaatccaggctgacgcatatattagtctcgtccttatcgcctcatctctctctctctctctctctctctctttctctctcgcctctctgtgtagcgcgcggcggtggtcgggcggcatcccggcgtaggcaacagtggtggcgagatgggaggcggtggcgcatcacagctattcactttagcgccttgaataataatcggcatcatgatctacttatgcttcgtgcaagggggaagattgtagatacacatggtcacagctaagtgctatgatcgcggctcatctctccaaatagattcatgccatccgtacttaaacagaaccttatgttccgtgcatcctttctgaagtcttgaaatgttttatcgatgttttaccactacgacccatctgtagggtgtctcgcatcccgtcttgtttatgtttttatgtgtgccagcgcaccattctagcacgcgctttgttcctgaacaaacaccttagccgtggtattaaatggaaatactgcatgatcatagcaggaactctcttcttcgaaggatgtccgtcaacatcacctaggtcatctcgtctaatcttatatcgtagtgccttacaaaccaggcgtgcttctaggttctcgtactcctcaacgcgatagaggatacaatcattcagacatacgtgtatcttctgtacttccagtctgagagggcagattaccattttagcttcgtacgttgtttcgagcaattcgtttccctcgggaagaatattctttacgagtttcaataactcgccaaatgccttgtcagtcacaccatattttgccttccattgtcagaattccagtgtgtcgacctgcaggcatgcaagcttggcactggccgtcgttttacaacgtcgtgactgggaaaaccctggcgttacccaacttaatcgccttgcagcacatccccctttcgccagctggcgtaatagcgaagaggcccgcaccgatcgcccttcccaacagttgcgcagcctgaatggcgaatgctagagcagcttgagcttggatcagattgtcgtttcccgccttcagtttaaactatcagtgtttgacaggatatattggcgggtaaacctaagagaaaagagcgtttattagaataatcggatatttaaaagggcgtgaaaaggtttatccgttcgtccatttgtatgtg

* Sequence corresponding to the HA-XA21 module is colored in red.
